# Treatment of Hip-Joint Disease

**Published:** 1904-05

**Authors:** 


### TREATMENT OF HIP-JOINT DISEASE.*

The successful treatment of hip-joint disease requires an accurate knowledge of the pathological conditions present in the case, varying from a slight ostitis, that tends to a perfect functional recovery, to an acute, infectious, destructive involvement of the entire joint structures and invading the surrounding tissues. It requires likewise knowledge of the function of the joint, a ball-and-socket joint permitting motion in all directions and placed at the junction of two segments of unequal size, its muscles and supplying nerves;

*Read by Wm. P. Mathews, M.D., Richmond, Va., Professor of Anatomy Medical College of Virginia, before the Richmond Academy of Medicine and Surgery, January 12, 1904.

also considerable mechanical skill and ingenuity, and intelligent co-operation on the part of the mother or nurse in the execution of the various details of treatment.

The object of treatment is to prevent the symptoms and the natural, i. e. untreated, effects of this disease, e. g., to relieve the pain, so annoying and depressing to the vitality; to overcome the muscular spasm that increases the intra-articular pressure and friction causing distortion and deformity; to prevent or correct deformity or distortion and by these efforts to prevent that irremediable condition of upward displacement of the femur.

In many advanced cases removal of all diseased parts is necessary even to amputation at the joint itself, on account of the severity of the case and its dangerous complications. The best method of treatment of hip-joint disease is the one that most effectively assures perfect rest, fixation and protection of the diseased joint, and this under the best possible hygienic surroundings. The repair must be effected by absorption, ejection or enclosure of the diseased processes. The granulation tissue may become sufficiently organized to resist the further infection of the tubercular bacilli and become solidified into fibrous tissue.

When the disease is limited to the articular extremity of the femur and has been recognized early, then treatment is very simple indeed, as perfect rest in bed with slight traction will almost invariably give a perfect functional result in from six to twelve months. I mean, of course, a portable bed, such as the Bradford and Lovett gas-pipe frame bed or the Phelps plaster-bed, either of which can be carried about easily and kept in the open air or sunshine as desired. If the case is further advanced and the joint invaded, then absolute rest, relief of all function, protection against jars, pressure and friction must be secured, which means that distraction of the opposed bony surfaces be secured and retained until the acute stages and symptoms shall have passed or subsided. This implies that the joint is practically a broken joint and is to be treated as a broken bone by splinting, stilting and traction. The patient should be put to bed on a narrow firm mattress, and the body secured by the Marsh method or else placed on the Lovett frame, the diseased limb elevated until the lumbar spine rests on the bed and traction applied in this line by the weight and pulley attachment. The weight should be the least that will relieve the muscular spasm and consequently the pain and pressure, varying from five to twenty pounds. The adhesive plaster—moleskin—should come up on the thigh nearly to the hip so as to produce distraction at the hip and not at the knee-joint. The footpiece should be just a little wider than the foot so as not to allow pressure on the malleoli. As the pain and spasm subside the limb is gradually lowered until its normal position is attained, usually in from two to six weeks. Sometimes longitudinal traction is not sufficient to secure this result and lateral traction has to be added; usually five pounds is enough.

When the acute symptoms have subsided and the deformity corrected, some one of the devices for ambulatory treatment may be applied, either the modified long Taylor or Phelps brace, or the Thomas iron splint, or as we use, the plaster spica bandage and crutches with the high shoe, two and a half inches, on the well foot.

The old traction hip splint was not intended as a fixation appliance by its inventor, Davis, who said, "the first splint, as well as all my modifications, admits of free motion of the diseased joint, but rigidly excludes all friction of the diseased surfaces upon one another." Actual distraction is that at which he aimed, but the splint did not secure it. We use the brace in ambulatory treatment because it is practically as good as any other method even if it does not immobilize the joint, and then only when the symptoms show that the disease is quiescent. Thomas splint aims at the perfect immobilization of the joint, and thus prevents friction and pressure of its surfaces.

Whitman says "this brace, so effective in preventing and overcoming flexion deformity, does not prevent lateral distortion." This can be prevented, however, by an additional lateral bar. We prefer and usually use a close, snug-fitting, properly-padded plaster spica bandage, including the lower half of the thorax and extending to the end of the toes. The joint is further protected by the addition of a piece of light steel or block tin extending from the middle of the back to the lower third of the thigh, another over the front of the joint and another at the back of the knee—all incorporated in the plaster. The antero-posterior support deserves special attention. This appliance permits the patient to get about with crutches and the high shoe as well as any other appliance, and as thoroughly protects and fixes the joint as any other known method of treatment. It is inexpensive, cleanly and easily applied.

If a brace is to be applied, the long hip splint with a short spica of the Lorenz pattern comes nearer to the ideal perfect combination. The time required for a complete cure of uncomplicated cases varies from two to four years, and it is better to continue for a year too long than for a week too short. The treatment is to be continued until all of the symptoms have entirely disappeared, when there is no muscular spasm, pain nor increasing limited motion and when weight-bearing causes no discomfort.

Abscess is the most common complication of this disease, occurring in about fifty per cent. of all the cases. This, of course, includes all of the liquid accumulations of the tuberculous processes either within the capsule or in the periarticular structures sufficient to form an appreciable tumor. The abscess first appears in the space between the sartorius and tensor fasciæ femoris muscles, or on the inner side of the thigh, or else beneath the gluteal muscles, varying with the point at which the capsule is ruptured, the weakest point being at the bursa of the ilio-psoas muscle.

Abscess is dangerous to the life of the patient on account of profuse suppuration and its consequences that may follow infection, and to the functional activity because of adhesions and contraction. Sometimes, and in perhaps the larger proportion of cases where the joint has been protected, the formation of an abscess is insidious, its appearance long delayed and, therefore, not recognized. Again, it appears early, due to the destructive involvement of the joint, and is accompanied by an acute exacerbation of all the symptoms with a decided temperature. The great danger is infection, which may occur before an opening forms, but as a rule the abscess is sterile until the skin is open. Two methods are now employed by good surgeons, the one class advocating absolute non-interference with the abscess cavity; the other class advocating immediate evacuation and thorough cleaning. About twenty per cent. disappear without treatment, so that it is wise to let the symptomatic abscess alone when proper after-treatment can not be secured.

Wherever asepsis can be secured free incision with complete evacuation of the contents of the abscess, careful exploration of the capsule of the joint and removal of any diseased foci that may be found in the interior of the capsule, and the proper closing of the wound, is the routine treatment of the majority of the orthopedic surgeons of the day. It relieves the system of the burden of absorption of a great mass of necrotic material, causes little or no disturbance and heals rapidly. If infection has occurred or the diseased foci can not be thoroughly removed so that further fluid accumulations may be expected, drainage must be established. The iodoform emulsion formerly so much in vogue has been thoroughly tested and discarded.

Sinuses should not be interfered with as a rule, until the active stages have passed, and when it is judicious to close them at all, complete dissection and thorough removal of the disease is the proper radical treatment. Sometimes the symptoms can not be controlled, as above indicated, when an *exploratory incision* becomes necessary, and this is usually done by an antero-lateral incision about three inches long, along the line of junction between the tensor fasciæ femoris and the gluteus medius muscles, which allows of free inspection and manipulation of the head of the femur after the joint has been opened in the line of the neck. This may be necessary in the later stages of the disease for long-continued suppuration or to remove the necrosed bone.

Again, excision of the hip may be found necessary in certain cases to save the life of the patient. Where there is progressive failure in health, or where infection can not be properly drained, or where there is extension of the disease to the shaft of the femur, or some such serious complication exists, this is the treatment of necessity. Koenig's method is probably the best, a description of which will be found in the usual text-books.

Amputation of the joint may be necessary even after an excision on account of continued suppuration and exhaustion, or from extension of the disease to the lower part of the shaft.

Some cases of untreated and even treated cases of hip-joint disease get well with a firm ankylosis with decided flexion deformity, which is best treated with linear osteotomy of the femur about two inches below the trochanters.

Bier's treatment of tuberculous joint disease is deserving of some attention, as it has been recently taken up by some of our leading orthopedic workers and is giving splendid results. The action of the venous or passive congestion is, according to Bier, as follows: 1. It increases the formation of fibrous tissue and induces hypertrophy of the bones. 2. It has a bactericidal action in infectious joint disease, notably tuberculosis. 3. It exercises an absorptive effect on the effused products of the disease and on new formations that check joint motion. 4. It relieves pain and lessens the activity of progressive disease.—*Virginia Medical Semi-Monthly.*

